# Supplementary material for: The Evolution and Characterization of the RNA Interference Pathways in Lophotrochozoa
Source: Genome Biol Evol. 2024 May 7;16(5):evae098. doi: 10.1093/gbe/evae098 (PMC11114477; doi:10.1093/gbe/evae098)
Supplement: evae098_Supplementary_Data [file evae098_supplementary_data.zip › Supplementary_materials.zip!/selected_miRNAs/NoGe_selected_miRNAs/mirdeep_pdf_files/NMRB01001254.1_Notospermus_geniculatus_strain_Ushimado_scaffold1254,_whole_genome_shotgun_sequence_26706.pdf]

Provisional ID : NMRB01001254.1_Notospermus_geniculatus_strain_Ushimado_scaffold1254,_whole_genome_shotgun_sequence_26706

Score total	:	47237.3
Score for star read(s)	:	3.9
Score for read counts	:	47229.3
Score for mfe	:	1.1
Score for randfold	:	0
Score for cons. seed	:	3
Total read count	:	92650
Mature read count	:	92152
Loop read count	:	0
Star read count	:	498

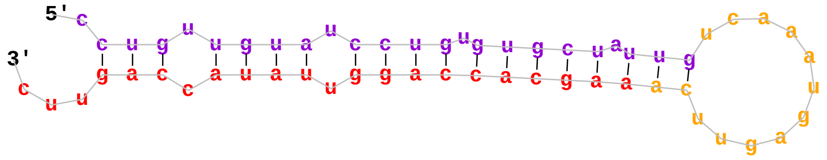

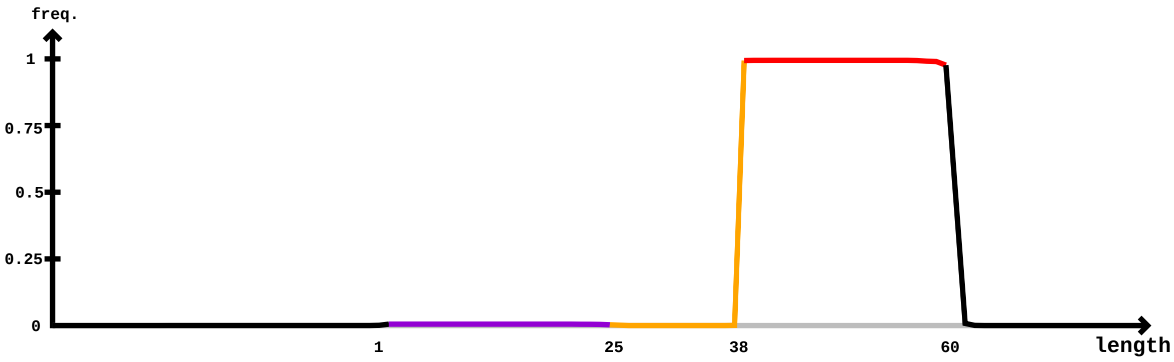

Star Mature

```
5'- uagaaguuguuuugucggauugcugcccagugcccuguuguauccugugugcuauugucaaaugaguucaaagcaccagguuauaccaguucauuuggcaugcaaauaaaua  -3'   obs
    uagaaguuguuuugucggauugcugcccagugcccuguuguauccugugugcuauugucaaaugaguucaaagcaccagguuauaccaguucauuuggcaugcaaauaaaua         exp
    ...................((((((((.((((..(((.((((.((((.(((((.(((...........)))))))))))).)))).)))..)))).)))).)))).......   reads    mm    sample
    ..............ucggauugcugccUagug................................................................................   1        1     Mm3
    .................gauuUcugcccagugccc.............................................................................   2        1     Mm3
    .................gauuUcugcccagugcccug...........................................................................   1        1     Mm3
    ................................cccuguuguauccugugu..............................................................   1        0     Mm3
    ................................cccuguuguauccugugugcu...........................................................   21       0     Mm3
    ................................cccuguuguauccugugCgcua..........................................................   1        1     Mm3
    ................................cccuguuguauccugugugcua..........................................................   2        0     Mm3
    ................................cccuguuguauccugugugcuU..........................................................   2        1     Mm3
    ................................cccuguuguauUcugugugcuau.........................................................   1        1     Mm3
    ................................cccuguuguauccugugugcuau.........................................................   16       0     Mm3
    ................................cccuguuguauccugugugcuauu........................................................   25       0     Mm3
    ................................cccuguuguauccugugugcuauug.......................................................   9        0     Mm3
    .................................ccuguuguauccugugugcu...........................................................   1        0     Mm3
    .................................ccuguuguauccugugugcua..........................................................   1        0     Mm3
    .................................ccuguuguauccugugugcuau.........................................................   5        0     Mm3
    .................................ccuguuguauccugugugcuauu........................................................   19       0     Mm3
    .................................ccuguuguauccugugugcuauA........................................................   1        1     Mm3
    .................................ccuguuguauccugugugcuauug.......................................................   45       0     Mm3
    .................................ccuguuguauccuguguCcuauug.......................................................   1        1     Mm3
    .................................ccuguuguauccugugGgcuauug.......................................................   1        1     Mm3
    .................................ccuguuguauccugugugcuauugA......................................................   1        1     Mm3
    .................................ccuguuguauccugugugcuauugG......................................................   1        1     Mm3
    .................................ccuguuguauccugugugcuauugucaaaugaguuca..........................................   1        0     Mm3
    .........................................................ucaaaugaguucaaagcaccagguu..............................   1        0     Mm3
    .....................................................................aaagcaccagguuauaccaguu.....................   2        0     Mm3
    .....................................................................Caagcaccagguuauaccaguuc....................   2        1     Mm3
    .....................................................................aaagcaccagguuauaccaguuc....................   1        0     Mm3
    .....................................................................Caagcaccagguuauaccaguuca...................   1        1     Mm3
    ......................................................................aagcaccagguuauacca........................   5        0     Mm3
    ......................................................................aagcaccagguuauaccagu......................   10       0     Mm3
    ......................................................................aagcaccagguuauaccaAuu.....................   1        1     Mm3
    ......................................................................aagcaccagguuauacAaguu.....................   1        1     Mm3
    ......................................................................aagcaccagguuauaccaguu.....................   104      0     Mm3
```

Star Mature

```
uagaaguuguuuugucggauugcugcccagugcccuguuguauccugugugcuauugucaaaugaguucaaagcaccagguuauaccaguucauuuggcaugcaaauaaaua
......................................................................aaUcaccagguuauaccaguuc....................  1     1  Mm3
......................................................................aagcaccagguuaAaccaguuc....................  1     1  Mm3
......................................................................aagcaccagguuauaccagCuc....................  1     1  Mm3
......................................................................Gagcaccagguuauaccaguuc....................  2     1  Mm3
......................................................................Cagcaccagguuauaccaguuc....................  1     1  Mm3
......................................................................aagcaccagguuauaUcaguuc....................  1     1  Mm3
......................................................................aagcaccagguuaCaccaguuc....................  2     1  Mm3
......................................................................aagcaccagguuauacGaguuc....................  13    1  Mm3
......................................................................aagcaNcagguuauaccaguuc....................  2     1  Mm3
......................................................................aaAcaccagguuauaccaguuc....................  1     1  Mm3
......................................................................aagcaccagguuauUccaguuc....................  2     1  Mm3
......................................................................aagcaccaUguuauaccaguuc....................  1     1  Mm3
......................................................................aagcaccaggGuauaccaguuc....................  6     1  Mm3
......................................................................aagcaccagguuauaccaguuU....................  9     1  Mm3
......................................................................aagcaccagguuauaccGguuc....................  21    1  Mm3
......................................................................aagcaccGgguuauaccaguuc....................  2     1  Mm3
......................................................................aagcaccaAguuauaccaguuc....................  1     1  Mm3
......................................................................aagcaccagguuauaccCguuc....................  24    1  Mm3
......................................................................aagcaccagUuuauaccaguuc....................  2     1  Mm3
......................................................................aagcacUagguuauaccaguuc....................  3     1  Mm3
......................................................................Uagcaccagguuauaccaguuc....................  4     1  Mm3
......................................................................aagcaccaggAuauaccaguuc....................  14    1  Mm3
......................................................................aagcaUcagguuauaccaguuc....................  1     1  Mm3
......................................................................aagAaccagguuauaccaguuc....................  1     1  Mm3
......................................................................aagcaccagguuauaccaguuc....................  5641  0  Mm3
......................................................................aagUaccagguuauaccaguuc....................  1     1  Mm3
......................................................................aagcaccagguuauacUaguuc....................  30    1  Mm3
......................................................................aagcaccagguuauaccaguCc....................  1     1  Mm3
......................................................................aagcaccagguuauGccaguuc....................  2     1  Mm3
......................................................................aagcaccUgguuauaccaguuc....................  2     1  Mm3
......................................................................Nagcaccagguuauaccaguuc....................  1     1  Mm3
......................................................................aagcaccaggCuauaccaguuc....................  7     1  Mm3
......................................................................aagcaccagguuauaccaguuA....................  3     1  Mm3
......................................................................aagcaccagguuauaccUguuc....................  9     1  Mm3
......................................................................aUgcaccagguuauaccaguuc....................  3     1  Mm3
......................................................................aagcaccagguuauaccaUuuc....................  1     1  Mm3
......................................................................aagcaccagguuauacAaguuc....................  21    1  Mm3
......................................................................aaAcaccagguuauaccaguuca...................  2     1  Mm3
......................................................................Uagcaccagguuauaccaguuca...................  2     1  Mm3
......................................................................aagcaccagguuauaccaguucC...................  4     1  Mm3
......................................................................aagcaccagguuauacGaguuca...................  1     1  Mm3
......................................................................aagcaccagguuauaccUguuca...................  4     1  Mm3
......................................................................aagcaccGgguuauaccaguuca...................  1     1  Mm3
......................................................................aagcaccaggCuauaccaguuca...................  1     1  Mm3
......................................................................aagcaccagguuauaccGguuca...................  6     1  Mm3
......................................................................aagcaccaggAuauaccaguuca...................  2     1  Mm3
......................................................................aagcaccagguuauacAaguuca...................  3     1  Mm3
......................................................................aagcaccagguuauaccCguuca...................  6     1  Mm3
......................................................................aagcaccagguuauaccaguuca...................  1147  0  Mm3
......................................................................aagcaccagguuauaccaguuAa...................  1     1  Mm3
......................................................................aagcacNagguuauaccaguuca...................  1     1  Mm3
......................................................................aagcaccaggGuauaccaguuca...................  2     1  Mm3
......................................................................aagcaUcagguuauaccaguuca...................  1     1  Mm3
......................................................................aagcaccagguuauaccaguucU...................  290   1  Mm3
......................................................................aagcaccagguuauacUaguuca...................  2     1  Mm3
......................................................................aagcaccagguuauaccaguucau..................  42    0  Mm3
......................................................................aagcaccagguuauaccaguucCu..................  1     1  Mm3
......................................................................aagcaccagguuauaccaguucUu..................  7     1  Mm3
......................................................................aagcaccagguuauaccaguucaA..................  27    1  Mm3
......................................................................aagcaccagguuauaccaguucaG..................  3     1  Mm3
......................................................................aagcaccagguuauaccaguucaAu.................  3     1  Mm3
......................................................................aagcaccagguuauaccaguucauu.................  7     0  Mm3
.......................................................................agcaccagguuauaccaguuc....................  7     0  Mm3
.......................................................................agcaccagguuauaccaguucaC..................  1     1  Mm3
........................................................................gcaccagguuauaccaguuc....................  1     0  Mm3
...........................................................................ccagguuauaccaguuca...................  1     0  Mm3

.................................ccuguuguauccugugugcu...........................................................  9     0  Fm3
.................................ccuguuguauccugugugcuau.........................................................  1     0  Fm3
.................................ccuguuguauccugugugcuauu........................................................  9     0  Fm3
```

```
                                 Star                                     Mature
uagaaguuguuuugucggauugcugcccagugcccuguuguauccugugugcuauugucaaaugaguucaaagcaccagguuauaccaguucauuuggcaugcaaauaaaua
.................................ccuguuguauccugugugcuauuU.......................................................  1     1  Fm3
.................................ccuguuguauccugugugcuauug.......................................................  55    0  Fm3
.....................................................................Caagcaccagguuauaccaguuc....................  1     1  Fm3
......................................................................aagcaccagguuauacca........................  19    0  Fm3
......................................................................aagcaccaUguuauaccag.......................  1     1  Fm3
......................................................................aGgcaccagguuauaccag.......................  1     1  Fm3
......................................................................aagcaccagguuauaccag.......................  195   0  Fm3
......................................................................aagcaccGgguuauaccag.......................  1     1  Fm3
......................................................................aagcaccagguuauaccaU.......................  5     1  Fm3
......................................................................aagcaccagguuauaccCg.......................  2     1  Fm3
......................................................................aagcaccagguuauacAag.......................  2     1  Fm3
......................................................................aagcaccagguuauaccagC......................  1     1  Fm3
......................................................................aagcaccagguuauaccUgu......................  1     1  Fm3
......................................................................Uagcaccagguuauaccagu......................  1     1  Fm3
......................................................................aagcaccagguuauaccagu......................  27    0  Fm3
......................................................................aagcaccagguuauaUcagu......................  3     1  Fm3
......................................................................aagcaccaggGuauaccaguu.....................  1     1  Fm3
......................................................................aagcaAcagguuauaccaguu.....................  1     1  Fm3
......................................................................aagcaccagguuauacAaguu.....................  2     1  Fm3
......................................................................aagcaccagguuauGccaguu.....................  1     1  Fm3
......................................................................aagcaccagguuauaccaguu.....................  334   0  Fm3
......................................................................aagcaccagguuauaccUguu.....................  2     1  Fm3
......................................................................aagcaccagguuauaccGguu.....................  2     1  Fm3
......................................................................aagcaccagguGauaccaguu.....................  2     1  Fm3
......................................................................aagcaccaggAuauaccaguu.....................  2     1  Fm3
......................................................................Gagcaccagguuauaccaguu.....................  1     1  Fm3
......................................................................aagcaccagguuauacGaguu.....................  1     1  Fm3
......................................................................aagcaccagguuauacUaguu.....................  2     1  Fm3
......................................................................aagAaccagguuauaccaguu.....................  1     1  Fm3
......................................................................aagcaccaggCuauaccaguuc....................  2     1  Fm3
......................................................................aagcaccaggAuauaccaguuc....................  9     1  Fm3
......................................................................aagcaccagguuauaccaguGc....................  1     1  Fm3
......................................................................aagGaccagguuauaccaguuc....................  2     1  Fm3
......................................................................aagcaccagguuauacGaguuc....................  2     1  Fm3
......................................................................aagcaccagguuauaccaguuU....................  5     1  Fm3
......................................................................aagcaccagguuauaccagAuc....................  2     1  Fm3
......................................................................aagcaccagguuauaccaguuG....................  1     1  Fm3
......................................................................aaCcaccagguuauaccaguuc....................  1     1  Fm3
......................................................................aagcacAagguuauaccaguuc....................  2     1  Fm3
......................................................................aagcaccagguuauaccaguuc....................  2489  0  Fm3
......................................................................aagcaccagguuauaccUguuc....................  12    1  Fm3
......................................................................aagcaccagguuauaUcaguuc....................  1     1  Fm3
......................................................................aagcaccagguuauaccCguuc....................  4     1  Fm3
......................................................................aagcaccagguuauaccGguuc....................  10    1  Fm3
......................................................................aagcaccagguuaAaccaguuc....................  1     1  Fm3
......................................................................aagUaccagguuauaccaguuc....................  1     1  Fm3
......................................................................aaAcaccagguuauaccaguuc....................  1     1  Fm3
......................................................................aaUcaccagguuauaccaguuc....................  1     1  Fm3
......................................................................Gagcaccagguuauaccaguuc....................  1     1  Fm3
......................................................................aagcaccaggGuauaccaguuc....................  3     1  Fm3
......................................................................aagcaccGgguuauaccaguuc....................  1     1  Fm3
......................................................................aCgcaccagguuauaccaguuc....................  1     1  Fm3
......................................................................aagcaccagguuaCaccaguuc....................  1     1  Fm3
......................................................................aagcaccagguuauacUaguuc....................  10    1  Fm3
......................................................................aagAaccagguuauaccaguuc....................  2     1  Fm3
......................................................................aagcaccagguuGuaccaguuc....................  1     1  Fm3
......................................................................aagcaccagguCauaccaguuc....................  2     1  Fm3
......................................................................aagcaccagguuauaccaguAc....................  1     1  Fm3
......................................................................aagcaccagguuauacAaguuc....................  12    1  Fm3
......................................................................aagcacGagguuauaccaguuca...................  1     1  Fm3
......................................................................aagcaccagguuauaccGguuca...................  1     1  Fm3
......................................................................aagcaccagguuauaccaguuca...................  400   0  Fm3
......................................................................aagcaccagguuauaccaguucC...................  3     1  Fm3
......................................................................aagcaccaggCuauaccaguuca...................  2     1  Fm3
......................................................................aagcaccaggGuauaccaguuca...................  1     1  Fm3
......................................................................aagcaccagguuauaccaguucU...................  74    1  Fm3
......................................................................aagcaccagguuauacGaguuca...................  1     1  Fm3
......................................................................aagcaccagguuauaccUguuca...................  1     1  Fm3
......................................................................aagcaccagguuauaccCguuca...................  1     1  Fm3
......................................................................aagcaccagguuauacAaguuca...................  3     1  Fm3
```

```
                                Star                                 Mature
uagaaguuguuuugucggauugcugcccagugcccuguuguauccugugugcuauugucaaaugaguucaaagcaccagguuauaccaguucauuuggcaugcaaauaaaua
......................................................................aagcaccaggAuauaccaguuca...................   2     1   Fm3
......................................................................aagcaccagguuauacGaguucau..................   1     1   Fm3
......................................................................aagcaccagguuauaccaguucaG..................   4     1   Fm3
......................................................................aagcaccagguuauaccaguucau..................   23    0   Fm3
......................................................................aagcaccagguuauaccaguucaA..................   15    1   Fm3
......................................................................aagcaccagguuauaccaguucUu..................   1     1   Fm3
......................................................................aagcaccagguuauaccaguucauu.................   2     0   Fm3
.......................................................................agcaccagguuauaccaguuc....................   1     0   Fm3

.................................ccuguuguauccuguguUcuau.........................................................   1     1   Mm1
.................................ccuguuguauccugugugcuau.........................................................   17    0   Mm1
.................................ccuguuguauccugugugcuauu........................................................   7     0   Mm1
.................................ccuguuguauGcugugugcuauug.......................................................   1     1   Mm1
.................................ccuguuguauccugugugcuauug.......................................................   44    0   Mm1
.................................ccuguuguauccuguguUcuauug.......................................................   2     1   Mm1
...................................uguuguauccugugugcuauug.......................................................   4     0   Mm1
......................................................................aagcaccagguuauacca........................   19    0   Mm1
......................................................................aagcaccagguuauaccag.......................   5     0   Mm1
......................................................................aagcaccagguuauaccCg.......................   1     1   Mm1
......................................................................aagcaccagguuauaccagu......................   6     0   Mm1
......................................................................aagcaccagguuauaccaguu.....................   255   0   Mm1
......................................................................aagcaccagguuauacGaguu.....................   1     1   Mm1
......................................................................aagcaccaggGuauaccaguu.....................   1     1   Mm1
......................................................................aagcaccagguuauacUaguu.....................   1     1   Mm1
......................................................................aagcaccagguuauaccUguu.....................   1     1   Mm1
......................................................................aagcaccagguuauacAaguu.....................   2     1   Mm1
......................................................................aagcaccagguuauaccCguu.....................   1     1   Mm1
......................................................................aagcaccagguuauaccGguu.....................   1     1   Mm1
......................................................................aagcacUagguuauaccaguu.....................   1     1   Mm1
......................................................................aagcacUagguuauaccaguuc....................   2     1   Mm1
......................................................................aagcaccaggGuauaccaguuc....................   5     1   Mm1
......................................................................aagcaccagAuuauaccaguuc....................   2     1   Mm1
......................................................................aagcaccagguuauaccCguuc....................   27    1   Mm1
......................................................................aagcaccagguuauaccaguuU....................   3     1   Mm1
......................................................................aagcaccagguCauaccaguuc....................   2     1   Mm1
......................................................................aagcGccagguuauaccaguuc....................   1     1   Mm1
......................................................................NagcaccagguuauaccaguuC....................   5     1   Mm1
......................................................................aagcaccagguuauacAaguuc....................   24    1   Mm1
......................................................................aagcaNcagguuauaccaguuc....................   1     1   Mm1
......................................................................aagcacNagguuauaccaguuc....................   1     1   Mm1
......................................................................aagcaccagguuauUccaguuc....................   3     1   Mm1
......................................................................aagcaccagguuauaccaUuuc....................   1     1   Mm1
......................................................................aagcaccagguuauGccaguuc....................   1     1   Mm1
......................................................................aGgcaccagguuauaccaguuc....................   1     1   Mm1
......................................................................aagcaccagguUuaccaguuc.....................   1     1   Mm1
......................................................................aagcaccaggCuauaccaguuc....................   6     1   Mm1
......................................................................Uagcaccagguuauaccaguuc....................   4     1   Mm1
......................................................................aaAcaccagguuauaccaguuc....................   3     1   Mm1
......................................................................aUgcaccagguuauaccaguuc....................   1     1   Mm1
......................................................................aagcaccagguuauaccGguuc....................   27    1   Mm1
......................................................................aagcaccaAgguuauaccaguuc...................   1     1   Mm1
......................................................................aagcaccUgguuauaccaguuc....................   1     1   Mm1
......................................................................aagcaccagguuauaccaguuc....................   6477  0   Mm1
......................................................................aagAaccagguuauaccaguuc....................   1     1   Mm1
......................................................................aagcaccagguuauacUaguuc....................   14    1   Mm1
......................................................................aagcaccagguuaAaccaguuc....................   4     1   Mm1
......................................................................aagcaccagguuauacGaguuc....................   11    1   Mm1
......................................................................aagcaccagguuauaccaguCc....................   1     1   Mm1
......................................................................aagcaccagguuauaccUguuc....................   21    1   Mm1
......................................................................aagcaccagguuauCccaguuc....................   1     1   Mm1
......................................................................aaUcaccagguuauaccaguuc....................   2     1   Mm1
......................................................................aagcaccaggAuauaccaguuc....................   13    1   Mm1
......................................................................aagcaccagguuGuaccaguuc....................   1     1   Mm1
......................................................................aCgcaccagguuauaccaguuc....................   1     1   Mm1
......................................................................aagcaccagguuauacGaguuca...................   2     1   Mm1
......................................................................aagcaccagguuauacAaguuca...................   1     1   Mm1
......................................................................aagcaccagguuauaccaguucC...................   36    1   Mm1
......................................................................aagcaccagguuauaccaguucU...................   249   1   Mm1
......................................................................aagcaccagguuauaccUguuca...................   1     1   Mm1
......................................................................aagcaccagguuauaccaguuca...................   434   0   Mm1
```

Star **Mature**

```
uagaaguuguuuugucggauugcugcccagugcccuguuguauccugugugcuauugucaaaugaguucaaagcaccagguuauaccaguucauuuggcaugcaaauaaaua
......................................................................aagcaccaggGuauaccaguuca...................   1   1   Mm1
......................................................................aagcaccagguuauacUaguuca...................   1   1   Mm1
......................................................................aagAaccagguuauaccaguuca...................   1   1   Mm1
......................................................................aagcaccaggAuauaccaguuca...................   1   1   Mm1
......................................................................aagcaccagguuauaccGguuca...................   4   1   Mm1
......................................................................aagcaccagguuauaccCguuca...................   1   1   Mm1
......................................................................aagcaccagguuauaccaguucaA..................   26  1   Mm1
......................................................................aagcaccagguuauaccaguucau..................   32  0   Mm1
......................................................................aagcaccagguuauaccaguucaG..................   6   1   Mm1
......................................................................aagcaccagguuauaccaguucauu.................   2   0   Mm1
.......................................................................agcaccagguuauaccaguuc....................   1   0   Mm1
.......................................................................agcaccagguuauaccaguucU...................   3   1   Mm1

.................gauuUcugcccagugccc.............................................................................   1   1   Mm2
.................gauuUcugcccagugcccu............................................................................   2   1   Mm2
................................cccuguuguauccugugugcu...........................................................   1   0   Mm2
................................cccuguuguauccugugugcuU..........................................................   1   1   Mm2
................................cccuguuguauccugugugcuau.........................................................   1   0   Mm2
................................cccuguuguauccugugugcuauug.......................................................   1   0   Mm2
.................................ccuguuguauccugugugcu...........................................................   1   0   Mm2
.................................ccuguuguauccugugugcua..........................................................   1   0   Mm2
.................................ccuguuguauccugugugcuau.........................................................   2   0   Mm2
.................................ccuguuguauccugugugcuauA........................................................   1   1   Mm2
.................................ccuguuguauccugugugcuauu........................................................   25  0   Mm2
.................................ccuUuuguauccugugugcuauug.......................................................   2   1   Mm2
.................................ccuguuguauccugugugcuauug.......................................................   57  0   Mm2
.................................ccuguuguauccugugugcuauugA......................................................   1   1   Mm2
.........................................................ucaaaugaguucaaagcaccagguu..............................   2   0   Mm2
.....................................................................aaagcaccagguuauaccaguuc....................   13  0   Mm2
.....................................................................Caagcaccagguuauaccaguuc....................   23  1   Mm2
.....................................................................Caagcaccagguuauaccaguuca...................   1   1   Mm2
......................................................................aagcaccagguuauacca........................   11  0   Mm2
......................................................................aagcaccagguuauaccaU.......................   1   1   Mm2
......................................................................aagcaccagguuauaccag.......................   10  0   Mm2
......................................................................aagcaccagguuauaccUg.......................   1   1   Mm2
......................................................................aagcaccagguuauaccUgu......................   1   1   Mm2
......................................................................aagcaccagguuauaccagu......................   36  0   Mm2
......................................................................aagcaccagguuauaccGguu.....................   1   1   Mm2
......................................................................aagcaccagguuauacAaguu.....................   4   1   Mm2
......................................................................aagcaccaggAuauaccaguu.....................   1   1   Mm2
......................................................................aagcaccagguuauaccCguu.....................   2   1   Mm2
......................................................................aagcaccagguuauaccUguu.....................   1   1   Mm2
......................................................................aagcaccagguuauacUaguu.....................   1   1   Mm2
......................................................................aagcaccagAuuauaccaguu.....................   1   1   Mm2
......................................................................aagcaccagguuauaccaguu.....................   346 0   Mm2
......................................................................aagcaccaggGuauaccaguu.....................   1   1   Mm2
......................................................................aagcaccagguuauaccaguCc....................   5   1   Mm2
......................................................................aCgcaccagguuauaccaguuc....................   2   1   Mm2
......................................................................aagcaccaCguuauaccaguuc....................   1   1   Mm2
......................................................................aagcaccagguuauacAaguuc....................   174 1   Mm2
......................................................................aUgcaccagguuauaccaguuc....................   9   1   Mm2
......................................................................aagcaccagguGauaccaguuc....................   3   1   Mm2
......................................................................aagcaccagUuuauaccaguuc....................   4   1   Mm2
......................................................................aagcaccagguuauaccagCuc....................   3   1   Mm2
......................................................................aagcaccagguuauaccaCuuc....................   1   1   Mm2
......................................................................aagcaccaUguuauaccaguuc....................   4   1   Mm2
......................................................................aagcaccagguuauacGaguuc....................   53  1   Mm2
......................................................................aagcaccagguuauaccaUuuc....................   5   1   Mm2
......................................................................aagcacAagguuauaccaguuc....................   1   1   Mm2
......................................................................aGgcaccagguuauaccaguuc....................   11  1   Mm2
......................................................................aagAaccagguuauaccaguuc....................   10  1   Mm2
......................................................................aagcaccaggCuauaccaguuc....................   39  1   Mm2
......................................................................aagcaccagguuauaccaguuA....................   8   1   Mm2
......................................................................aagcaccagguuUuaccaguuc....................   6   1   Mm2
......................................................................aagcaccagguuauaccaAuuc....................   3   1   Mm2
......................................................................aagcaccagguAauaccaguuc....................   3   1   Mm2
......................................................................aagGaccagguuauaccaguuc....................   2   1   Mm2
......................................................................aagcaccagguuauaccUguuc....................   112 1   Mm2
......................................................................aagcaccCgguuauaccaguuc....................   1   1   Mm2
......................................................................aagcaccagguuauaccagAuc....................   5   1   Mm2
```

Star **Mature**

uagaaguuguuuugucggauugcugcccagugcccuguuguauccugugugcuauugucaaaugaguucaaagcaccagguuauaccaguucauuuggcaugcaaauaaaua			
....Gagcaccagguuauaccaguuc....................	8	1	Mm2
....aagUaccagguuauaccaguuc....................	6	1	Mm2
....aagcacNagguuauaccaguuc....................	2	1	Mm2
....aagcaccagguuauaccaguuc....................	39840	0	Mm2
....aaUcaccagguuauaccaguuc....................	9	1	Mm2
....aaCcaccagguuauaccaguuc....................	4	1	Mm2
....aagcaccagguuaAaccaguuc....................	8	1	Mm2
....aagcaccGgguuauaccaguuc....................	23	1	Mm2
....Uagcaccagguuauaccaguuc....................	21	1	Mm2
....aagcaccagguuauaccaguAc....................	1	1	Mm2
....aagcaccagguuaGaccaguuc....................	1	1	Mm2
....aagcaccagguuauaccCguuc....................	134	1	Mm2
....aagcaccagguuauacUaguuc....................	101	1	Mm2
....aaAcaccagguuauaccaguuc....................	9	1	Mm2
....aagcaccagguuauaUcaguuc....................	9	1	Mm2
....aagcaccagguuGuaccaguuc....................	4	1	Mm2
....aagcaccagguuauaccaguuU....................	22	1	Mm2
....aagcUccagguuauaccaguuc....................	1	1	Mm2
....aagcacUagguuauaccaguuc....................	13	1	Mm2
....aagcGccagguuauaccaguuc....................	4	1	Mm2
....aagcaccUgguuauaccaguuc....................	5	1	Mm2
....aagcaccagAuuauaccaguuc....................	11	1	Mm2
....aagcaccagguuauGccaguuc....................	4	1	Mm2
....aagcaccagguuaCaccaguuc....................	8	1	Mm2
....aagcaccaggGuauaccaguuc....................	50	1	Mm2
....aagcCccagguuauaccaguuc....................	3	1	Mm2
....aagcaNcagguuauaccaguuc....................	5	1	Mm2
....Cagcaccagguuauaccaguuc....................	3	1	Mm2
....aagcaccagguuauaccGguuc....................	111	1	Mm2
....aagcaccagguCauaccaguuc....................	1	1	Mm2
....aagcaccagguuauUccaguuc....................	7	1	Mm2
....aagcaccagCuuauaccaguuc....................	1	1	Mm2
....Nagcaccagguuauaccaguuc....................	21	1	Mm2
....aagcaccagguuCuaccaguuc....................	2	1	Mm2
....aagcaGcagguuauaccaguuc....................	1	1	Mm2
....aagcaccaggAuauaccaguuc....................	77	1	Mm2
....aagcaUcagguuauaccaguuc....................	9	1	Mm2
....aagcacUagguuauaccaguuca...................	1	1	Mm2
....aagcaccGgguuauaccaguuca...................	1	1	Mm2
....aaAcaccagguuauaccaguuca...................	1	1	Mm2
....aagcaccagguuauaccGguuca...................	9	1	Mm2
....aagcaccagguuauaccaguucU...................	1552	1	Mm2
....aagcaccagguuauacUaguuca...................	2	1	Mm2
....aagcaccaggAuauaccaguuca...................	5	1	Mm2
....aagcaccagguuauaccaguuca...................	2451	0	Mm2
....aagcaccagguuauaccCguuca...................	7	1	Mm2
....aagcacNagguuauaccaguuca...................	1	1	Mm2
....aagcaccagguuauaUcaguuca...................	1	1	Mm2
....aagcaccagguuauacAaguuca...................	13	1	Mm2
....aagcaccaggGuauaccaguuca...................	2	1	Mm2
....aagcaccagguuauaccaguuUa...................	1	1	Mm2
....aagcaccaggCuauaccaguuca...................	5	1	Mm2
....aagcaccagguuauaccUguuca...................	7	1	Mm2
....Nagcaccagguuauaccaguuca...................	1	1	Mm2
....aagcaccagguuauacGaguuca...................	4	1	Mm2
....aagcaNcagguuauaccaguuca...................	2	1	Mm2
....aagcaccagguuauaccaguucC...................	67	1	Mm2
....aagcaccagguuauaccGguucau..................	1	1	Mm2
....aagcaccagguuauaccaguucaA..................	112	1	Mm2
....aagcaccagguuauaccaguucau..................	76	0	Mm2
....aagcaccagguuauUccaguucau..................	1	1	Mm2
....aagcaccagguuauaccaguucaC..................	10	1	Mm2
....aGgcaccagguuauaccaguucau..................	1	1	Mm2
....aagcaccagguuauaccUguucau..................	1	1	Mm2
....aagcaccagguuauaccaguucaG..................	10	1	Mm2
....aagcaccagguuauaccaguucUu..................	38	1	Mm2
....aagcaAcagguuauaccaguucau..................	1	1	Mm2
....aagcaccagguuauaccaguucauu.................	11	0	Mm2
....aagcaccagguuauaccaguucaCu.................	2	1	Mm2
....aagcaccagguuauaccaguucUuu.................	3	1	Mm2

```
                                 Star                                  Mature
uagaaguuguuuugucggauugcugcccagugcccuguuguauccugugugcuauugucaaaugaguucaaagcaccagguuauaccaguucauuuggcaugcaaauaaaua
......................................................................aagcaccagguuauaccaguucaAu.................  7     1  Mm2
......................................................................aagcaccagguuauaccaguucauuu................  1     0  Mm2
.......................................................................agcaccagguuauaccaguu.....................  1     0  Mm2
.......................................................................agcaccagguuauaccaguuc....................  26    0  Mm2
.......................................................................aAcaccagguuauaccaguuc....................  1     1  Mm2
.......................................................................agcacUagguuauaccaguuc....................  1     1  Mm2
.......................................................................agcaccagguuauaAcaguuca...................  1     1  Mm2
.......................................................................agcaccagguuauaccaguucU...................  2     1  Mm2
........................................................................gcaccagguuauaccaguu.....................  1     0  Mm2
.........................................................................caccagguuauaccaguuc....................  1     0  Mm2

.................................ccuguuguauccugugugcu...........................................................  3     0  Fm2
.................................ccuguuguauAcugugugcuauu........................................................  1     1  Fm2
.................................ccuguuguauccugugugcuauu........................................................  22    0  Fm2
.................................ccuguuguauUcugugugcuauu........................................................  1     1  Fm2
.................................ccuguuguauccuguguAcuauug.......................................................  1     1  Fm2
.................................ccuguuguauccugugugcuauug.......................................................  34    0  Fm2
.....................................................................aaagcaccagguuauaccaguuc....................  8     0  Fm2
.....................................................................Caagcaccagguuauaccaguuc....................  2     1  Fm2
......................................................................aagcaccagguuauaccaUu......................  2     1  Fm2
......................................................................aagcaccagguuauaccagu......................  4     0  Fm2
......................................................................aagcaccagguuauaccCguu.....................  1     1  Fm2
......................................................................aagcaccagguuaAaccaguu.....................  1     1  Fm2
......................................................................aagcaccagguuauaccaguu.....................  78    0  Fm2
......................................................................aagcaccagguuauaccGguuc....................  34    1  Fm2
......................................................................aagcGccagguuauaccaguuc....................  1     1  Fm2
......................................................................aagcaccagguuUuaccaguuc....................  1     1  Fm2
......................................................................aagcaccaggCuauaccaguuc....................  16    1  Fm2
......................................................................aagcacAagguuauaccaguuc....................  1     1  Fm2
......................................................................aagcacNagguuauaccaguuc....................  1     1  Fm2
......................................................................aagcaccagguuauaccaguuA....................  11    1  Fm2
......................................................................aagcaccCgguuauaccaguuc....................  1     1  Fm2
......................................................................aagcaccagguuGuaccaguuc....................  1     1  Fm2
......................................................................Uagcaccagguuauaccaguuc....................  5     1  Fm2
......................................................................Gagcaccagguuauaccaguuc....................  2     1  Fm2
......................................................................aagcaccaggAuauaccaguuc....................  19    1  Fm2
......................................................................aagcaccagguuauaccagCuc....................  2     1  Fm2
......................................................................aagcaccagguuauacGaguuc....................  9     1  Fm2
......................................................................aagcaccGgguuauaccaguuc....................  6     1  Fm2
......................................................................aagcaccagguuauacUaguuc....................  28    1  Fm2
......................................................................aagAaccagguuauaccaguuc....................  1     1  Fm2
......................................................................Nagcaccagguuauaccaguuc....................  6     1  Fm2
......................................................................aUgcaccagguuauaccaguuc....................  1     1  Fm2
......................................................................aagcaccagguuauaccaguuG....................  1     1  Fm2
......................................................................aagcaccagguuauaUcaguuc....................  1     1  Fm2
......................................................................aGgcaccagguuauaccaguuc....................  2     1  Fm2
......................................................................aagcaNcagguuauaccaguuc....................  1     1  Fm2
......................................................................aagcaccagguuauacAaguuc....................  31    1  Fm2
......................................................................aagcaccagguGauaccaguuc....................  1     1  Fm2
......................................................................aagcaccagguuauaccaguGc....................  1     1  Fm2
......................................................................aagcaccaggGuauaccaguuc....................  6     1  Fm2
......................................................................aagcaUcagguuauaccaguuc....................  1     1  Fm2
......................................................................aagcaccagguuaAaccaguuc....................  1     1  Fm2
......................................................................aagcaccagguuaCaccaguuc....................  2     1  Fm2
......................................................................aagcaccagguuauGccaguuc....................  1     1  Fm2
......................................................................aagcaccagguuauaccagAuc....................  2     1  Fm2
......................................................................aagcaccagguuauaccCguuc....................  30    1  Fm2
......................................................................aagcaccagguuauaccUguuc....................  22    1  Fm2
......................................................................aagcaccaCguuauaccaguuc....................  1     1  Fm2
......................................................................aagcaccagguuauaccaUuuc....................  1     1  Fm2
......................................................................aagcCccagguuauaccaguuc....................  1     1  Fm2
......................................................................aagcaccagUuuauaccaguuc....................  1     1  Fm2
......................................................................aagcacUagguuauaccaguuc....................  2     1  Fm2
......................................................................aagcaccagguuauUccaguuc....................  1     1  Fm2
......................................................................aagcaccagguuauaccaguuc....................  8938  0  Fm2
......................................................................aagcaccagguuauacGaguuca...................  1     1  Fm2
......................................................................aagcaccagguuauaccaguAca...................  1     1  Fm2
......................................................................aagcaccagguuauaccaguucU...................  491   1  Fm2
......................................................................aagAaccagguuauaccaguuca...................  1     1  Fm2
......................................................................aagcaccagguuauaccagCuca...................  2     1  Fm2
```

Star **Mature**

```
uagaaguuguuuugucggauugcugcccagugcccuguuguauccugugugcuauugucaaaugaguucaaagcaccagguuauaccaguucauuuggcaugcaaauaaaua
......................................................................aagcaccagguuauaccaguuAa...................   2      1   Fm2
......................................................................aagcaccagguuauacAaguuca...................   4      1   Fm2
......................................................................aagcaccagguuauacUaguuca...................   5      1   Fm2
......................................................................aagcaccagguuauaccGguuca...................   1      1   Fm2
......................................................................aagcaccaggAuauaccaguuca...................   1      1   Fm2
......................................................................aagcaccaAguuauaccaguuca...................   1      1   Fm2
......................................................................aagcaccagguuGuaccaguuca...................   1      1   Fm2
......................................................................aagcaccagguuauaccaguucC...................   35     1   Fm2
......................................................................aCgcaccagguuauaccaguuca...................   1      1   Fm2
......................................................................aagcaccagguuauaccCguuca...................   5      1   Fm2
......................................................................aagcaccagguuauaccUguuca...................   4      1   Fm2
......................................................................Nagcaccagguuauaccaguuca...................   1      1   Fm2
......................................................................aagcaccagguuauaccaguuca...................   940    0   Fm2
......................................................................aagcaccagguuauaccaAuuca...................   1      1   Fm2
......................................................................aaAcaccagguuauaccaguuca...................   1      1   Fm2
......................................................................aagcaccagguuauaccaguucaA..................   5      1   Fm2
......................................................................aagcaccagguuauaccaguucUu..................   11     1   Fm2
......................................................................aagcaccagguuauaccGguucau..................   1      1   Fm2
......................................................................aagcaccagguuauaccaguucau..................   58     0   Fm2
......................................................................aagcaccagguuauaccCguucau..................   1      1   Fm2
......................................................................aagcaccagAuuauaccaguucau..................   1      1   Fm2
......................................................................aagcaccagguuauacAaguucau..................   2      1   Fm2
.......................................................................agcaccagguuauaccaguuc....................   3      0   Fm2
.......................................................................agcaccagguuauaccaguucU...................   5      1   Fm2
.........................................................................caccagguuauaccaguuc....................   3      0   Fm2

...........uugucggauugcugcccagugc...............................................................................   5      0   Fm1
.................................ccuguuguauccuguguAcu...........................................................   1      1   Fm1
.................................ccuguuguauccugugugcu...........................................................   1      0   Fm1
.................................ccuguuguauccugugugcuau.........................................................   2      0   Fm1
.................................ccuguuguauccugugugcuauu........................................................   14     0   Fm1
.................................ccuguuguauccugugugcuauug.......................................................   17     0   Fm1
.................................ccuguuguauccugugugcuCuug.......................................................   1      1   Fm1
.................................ccuguCguauccugugugcuauug.......................................................   1      1   Fm1
.........................................................ucaaaugaguucaaagcaccagguuauaccagu......................   1      0   Fm1
.........................................................ucaaaugaguucaaagcaccagguuauaccaguuc....................   2      0   Fm1
.....................................................................Caagcaccagguuauaccaguuc....................   6      1   Fm1
.....................................................................aaagcaccagguuauaccaguuc....................   6      0   Fm1
......................................................................aagcaccagguuauacca........................   13     0   Fm1
......................................................................aagcaccagguuauaccag.......................   2      0   Fm1
......................................................................aagcaccagguuauaccagu......................   13     0   Fm1
......................................................................aagcaccagguuauaccGguu.....................   1      1   Fm1
......................................................................aagcaccagguuauaccUguu.....................   1      1   Fm1
......................................................................aagcaNcagguuauaccaguu.....................   1      1   Fm1
......................................................................aagcaccagguuauaccaguu.....................   92     0   Fm1
......................................................................aagcaccagguuauacAaguu.....................   2      1   Fm1
......................................................................aUgcaccagguuauaccaguuc....................   3      1   Fm1
......................................................................aagAaccagguuauaccaguuc....................   8      1   Fm1
......................................................................aagcaccagguuaGaccaguuc....................   1      1   Fm1
......................................................................aaUcaccagguuauaccaguuc....................   3      1   Fm1
......................................................................aagcaccGgguuauaccaguuc....................   17     1   Fm1
......................................................................Nagcaccagguuauaccaguuc....................   5      1   Fm1
......................................................................aagcaccagguCauaccaguuc....................   2      1   Fm1
......................................................................aagcaccagguuauaccUguuc....................   37     1   Fm1
......................................................................aagUaccagguuauaccaguuc....................   3      1   Fm1
......................................................................aagGaccagguuauaccaguuc....................   1      1   Fm1
......................................................................aagcaccagguuauaccaUuuc....................   3      1   Fm1
......................................................................aagcaccaUguuauaccaguuc....................   2      1   Fm1
......................................................................aagcaccagguuauaccaguuc....................   13386  0   Fm1
......................................................................aagcaNcagguuauaccaguuc....................   4      1   Fm1
......................................................................aCgcaccagguuauaccaguuc....................   1      1   Fm1
......................................................................aagcaccagguuauaccCguuc....................   33     1   Fm1
......................................................................aGgcaccagguuauaccaguuc....................   2      1   Fm1
......................................................................aagcUccagguuauaccaguuc....................   2      1   Fm1
......................................................................aagcaccagguuauUccaguuc....................   1      1   Fm1
......................................................................aagcaUcagguuauaccaguuc....................   4      1   Fm1
......................................................................Uagcaccagguuauaccaguuc....................   9      1   Fm1
......................................................................aagcGccagguuauaccaguuc....................   3      1   Fm1
......................................................................aagcaccagguuauaccaguuU....................   12     1   Fm1
......................................................................aagcaccagguuauaccaguuA....................   10     1   Fm1
```

```
                                 Star                    Mature
uagaaguuguuuugucggauugcugcccagugcccuguuguauccugugugcuauugucaaaugaguucaaagcaccagguuauaccaguucauuuggcaugcaaauaaaua
......................................................................Gagcaccagguuauaccaguuc....................   5     1   Fm1
......................................................................aagcaccagguuauacUaguuc....................   32    1   Fm1
......................................................................aagcaccagguGauaccaguuc....................   2     1   Fm1
......................................................................aagcaccaggAuauaccaguuc....................   31    1   Fm1
......................................................................aagcaccagguuauGccaguuc....................   6     1   Fm1
......................................................................aagcaccaggGuauaccaguuc....................   19    1   Fm1
......................................................................aagcaccagguuauaccaguAc....................   1     1   Fm1
......................................................................aagcaccagUuuauaccaguuc....................   1     1   Fm1
......................................................................aagcaccagguuauaccGguuc....................   54    1   Fm1
......................................................................aagcaccaggCuauaccaguuc....................   16    1   Fm1
......................................................................aagcacAagguuauaccaguuc....................   3     1   Fm1
......................................................................aagcaccagguuauaccaguGc....................   1     1   Fm1
......................................................................aagcaccagguuUuaccaguuc....................   1     1   Fm1
......................................................................aagcaccagguuGuaccaguuc....................   1     1   Fm1
......................................................................aaCcaccagguuauaccaguuc....................   6     1   Fm1
......................................................................aagcaccagguuauacGaguuc....................   25    1   Fm1
......................................................................aaAcaccagguuauaccaguuc....................   3     1   Fm1
......................................................................aagcacUagguuauaccaguuc....................   5     1   Fm1
......................................................................aagcaccaCguuauaccaguuc....................   1     1   Fm1
......................................................................aagcaccagguuauacAaguuc....................   62    1   Fm1
......................................................................aagcaccagguuauaUcaguuc....................   4     1   Fm1
......................................................................aagcaccUgguuauaccaguuc....................   1     1   Fm1
......................................................................aagcCccagguuauaccaguuc....................   2     1   Fm1
......................................................................aagcUccagguuauaccaguuca...................   1     1   Fm1
......................................................................aagcaNcagguuauaccaguuca...................   1     1   Fm1
......................................................................aagcaccaggAuauaccaguuca...................   4     1   Fm1
......................................................................aagcaccagguuauaccaguucC...................   85    1   Fm1
......................................................................aagcaccagAuuauaccaguuca...................   1     1   Fm1
......................................................................aagcaccagguuauaccGguuca...................   1     1   Fm1
......................................................................aagcaccagguuauacUaguuca...................   7     1   Fm1
......................................................................aagcaccagguuauaccagAuca...................   1     1   Fm1
......................................................................aagcaccagguuauaccaguucU...................   745   1   Fm1
......................................................................aagcaccagguuauaccCguuca...................   5     1   Fm1
......................................................................aagcaccagguuauGccaguuca...................   1     1   Fm1
......................................................................aagcaccGgguuauaccaguuca...................   1     1   Fm1
......................................................................aagcaccagguuauacGaguuca...................   5     1   Fm1
......................................................................aagcaccagguuauacAaguuca...................   5     1   Fm1
......................................................................aagcaccagguuaCaccaguuca...................   1     1   Fm1
......................................................................aagcaccagguuauaccUguuca...................   7     1   Fm1
......................................................................aagcaccagguuauaccaguuca...................   1292  0   Fm1
......................................................................aagcaccagguuauaccaguucUu..................   49    1   Fm1
......................................................................aagcaccagguuauaccaguucaG..................   5     1   Fm1
......................................................................aagcaccagguuauaccaguucaA..................   38    1   Fm1
......................................................................aagcaccagguuauaccUguucau..................   1     1   Fm1
......................................................................aagcaccagguuauaccaguucau..................   72    0   Fm1
......................................................................aagcaccagguuauaccaguucCu..................   5     1   Fm1
......................................................................aagcaccagguuauaccaguuUau..................   1     1   Fm1
......................................................................aagcaccagguuauacUaguucau..................   1     1   Fm1
......................................................................aagcaccagguuauaccaguucaC..................   8     1   Fm1
......................................................................aagcaccagguuauaccaguucUuu.................   8     1   Fm1
......................................................................aagcaccagguuauaccaguucauu.................   5     0   Fm1
......................................................................aagcaccagguuauaccaguucauA.................   2     1   Fm1
......................................................................aagcaccagguuauaccaguucaAu.................   1     1   Fm1
......................................................................aagcaccagguuauaccaguucaCu.................   1     1   Fm1
.......................................................................agcaccagguuauaccaguuc....................   2     0   Fm1
```
